# Advanced disease and biochemical phenotype shift in multiple endocrine neoplasia type 2A-related pheochromocytoma

**DOI:** 10.1210/jcemcr/luag038

**Published:** 2026-03-12

**Authors:** Juro Yanagida, Kiyomi Horiuchi, Yusaku Yoshida, Tomoko Yamamoto

**Affiliations:** Department of Endocrine Surgery, Tokyo Women's Medical University Hospital, Shinjuku-ku, Tokyo 162-8666, Japan; Department of Endocrine Surgery, Tokyo Women's Medical University Hospital, Shinjuku-ku, Tokyo 162-8666, Japan; Department of Endocrine Surgery, Tokyo Women's Medical University Hospital, Shinjuku-ku, Tokyo 162-8666, Japan; Department of Diagnostic Pathology, Tokyo Women's Medical University Hospital, Shinjuku-ku, Tokyo 162-8666, Japan

**Keywords:** multiple endocrine neoplasia type 2, pheochromocytoma, distant metastasis, biochemical phenotype

## Abstract

The incidence of metastatic recurrence in multiple endocrine neoplasia type 2 (MEN2)-related pheochromocytoma is low, and reports are scarce. Moreover, there are no reports detailing changes in biochemical findings at the time of metastatic recurrence. We describe here the case of a woman in her 40s with MEN2-related pheochromocytoma. She exhibited an increase in spot urine normetanephrine levels 2 years and 8 months after laparoscopic right adrenalectomy for right pheochromocytoma, leading to the diagnosis of peritoneal dissemination and distant metastasis. She underwent chemotherapy, which was ineffective, and died 3 years and 2 months after the initial surgery. Metastasis and recurrence of MEN2-related pheochromocytoma are rare, and a change in the biochemical phenotype is also uncommon. Therefore, we report the clinical course of this case in detail.

## Introduction

Multiple endocrine neoplasia type 2 (MEN2) is an autosomal dominant hereditary disorder that can cause medullary thyroid carcinoma, pheochromocytoma, and hyperparathyroidism, with pheochromocytoma occurring in approximately 50% of MEN2 cases [1]. All pheochromocytomas have been shown to have the potential for metastasis and recurrence during long-term follow-up [2]. Therefore, in the fifth edition of the World Health Organization classification (2022), pheochromocytoma is considered a malignant tumor with the potential to metastasize or recur [3]. A recent meta-analysis also found a higher frequency of hereditary pheochromocytoma (33.8%) than previously believed [4]. In hereditary pheochromocytomas, the frequency of metastasis and recurrence is known to vary depending on the causative gene, and is rare in MEN2 with rearranged during transfection (*RET*) gene pathogenic variant (2.9%) [5]. Here, we report a rare case of MEN2A-related pheochromocytoma that developed multiple metastases and resulted in death about 3 years after surgery. For risk stratification, the grading of adrenal pheochromocytoma and paraganglioma (GAPP) score was assessed retrospectively at the time of manuscript preparation [6].

## Case presentation

**Figure 1:**
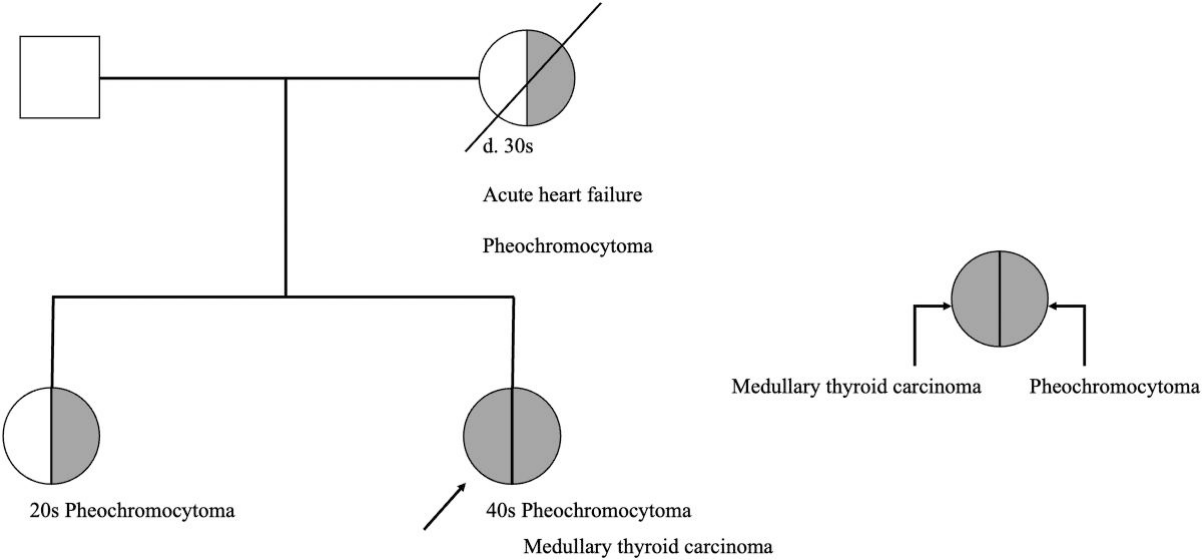
The patient's family tree. Her mother and sister had pheochromocytoma. Squares in the figure indicate males and circles indicate females. Diagonal slashes denote deceased individuals. Shaded areas within the symbols represent affected organs: Shading on the right side = pheochromocytoma; Shading on the left side = medullary thyroid carcinoma. Our patient (indicated by the arrow) was in her 40s and developed both medullary thyroid carcinoma and pheochromocytoma. d., died.

A woman in her 40s with no significant medical history presented to our hospital with the chief complaint of palpitations and neck discomfort. Her mother had died in her 30s of acute heart failure, and an autopsy had revealed pheochromocytoma. Her older sister had a history of adrenalectomy for pheochromocytoma in her 20s, with confirmation of the germline pathogenic variant in the *RET* gene codon 634 (Fig. 1). Based on the patient's family history, medullary thyroid carcinoma and pheochromocytoma were suspected, and a comprehensive diagnostic work-up was initiated.

## Diagnostic assessment

**Figure 2:**
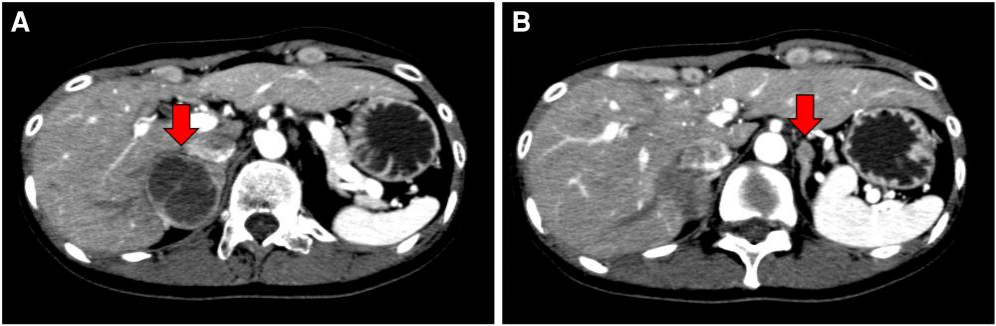
CT findings. A 4.6 × 3.8 cm mass with cystic changes was observed in the right adrenal gland (A), and a 1.2 × 1.2 cm solid mass was observed in the left adrenal gland (B).

**Figure 3:**
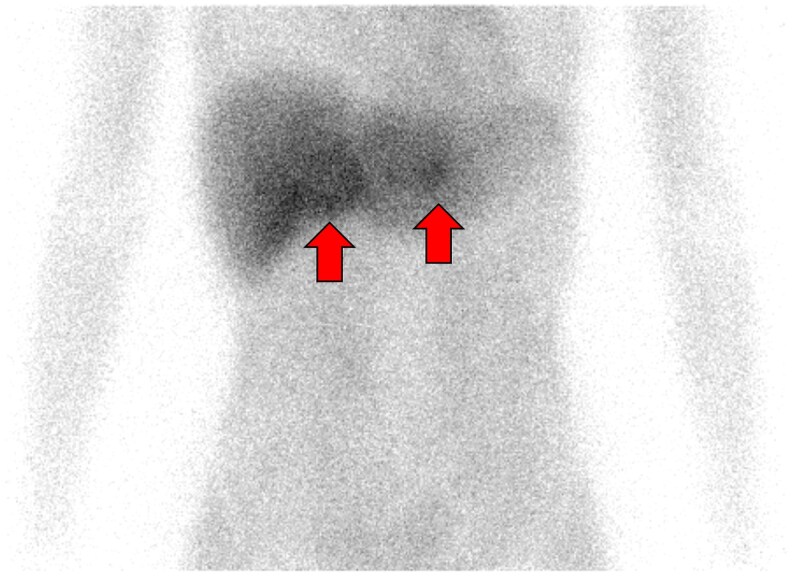
^123^I-meta-iodobenzylguanidine (MIBG) scintigraphy. Enhanced accumulation of MIBG in the adrenal masses was observed bilaterally.

Blood tests in our patient showed elevated blood levels of adrenaline 169 pg/mL (SI: 922 pmol/L) (reference range, <100 pg/mL [SI: <545 pmol/L]), noradrenaline 1406 pg/mL (SI: 8315 pmol/L) (reference range, 100-450 pg/mL [SI: 591-2661 pmol/L]), and dopamine 21 pg/mL (SI: 137 pmol/L) (reference range, <20 pg/mL [SI: <130 pmol/L]). Her carcinoembryonic antigen (CEA) level was 3.7 ng/mL (SI: 3.7 μg/L) (reference range, <5.0 ng/mL [SI: <5.0 μg/L]), and calcitonin was markedly elevated at 260 pg/mL (SI: 75.9 pmol/L) (reference range, 17.1-58.7 pg/mL [SI: 5.0-17.1 pmol/L]). A 24-hour urine analysis showed elevated levels of metanephrine (MN) 1850 μg/day (SI: 9379.5 nmol/day) (reference range, 40-190 μg/day [SI: 203-964 nmol/day]) and normetanephrine (NMN) 2720 μg/day (SI: 14 851 nmol/day) (reference range, 90-330 μg/day [SI: 491-1802nmol/day]), indicating an adrenergic biochemical phenotype. Ultrasonography of the neck showed an irregular mass measuring 0.8 × 0.8 cm in the right lobe of the thyroid gland, and 0.5 × 0.3 cm in the left lobe. Abdominal computed tomography (CT) showed a 4.6 × 3.8 cm mass with cystic changes in the right adrenal gland, and a 1.2 × 1.2 cm solid mass in the left adrenal gland. The unenhanced CT attenuation was 39.8 Hounsfield units (HU) for the right adrenal mass and 44.7 HU for the left adrenal mass. There was no evidence of invasion of the surrounding tissues by either mass (Fig. 2). ^123^I-meta-iodobenzylguanidine (MIBG) scintigraphy showed increased accumulation in the adrenal masses bilaterally. There was no accumulation in distant organs (Fig. 3). Based on the above, the patient was diagnosed with MEN2A-related bilateral adrenal pheochromocytoma (cT1N0M0, cStage I), and medullary thyroid carcinoma (cT1aN0M0, cStage I). We decided to prioritize surgical resection of the pheochromocytoma.

## Treatment

**Figure 4:**
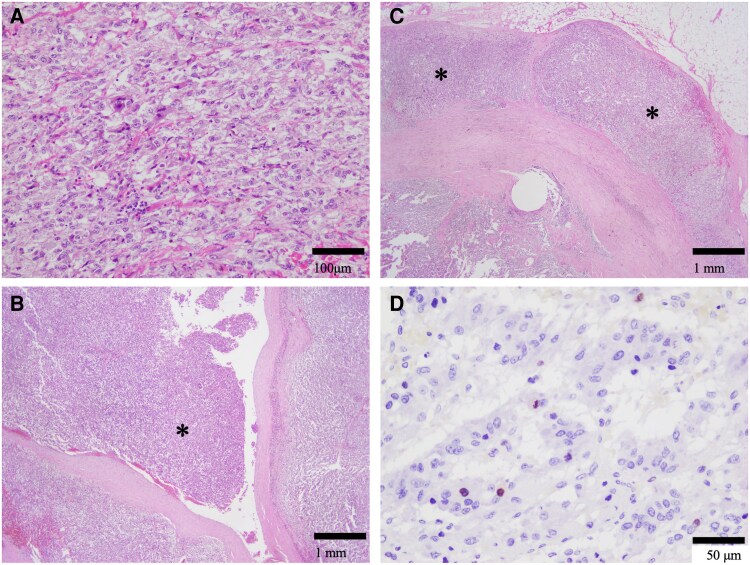
Histopathological findings. Hematoxylin–eosin (HE) staining. (A) The tumor cells proliferated to form small nests. (B) Venous invasion (*). (C) Partial extracapsular invasion (*). (D) Ki-67 immunohistochemistry demonstrated a labeling index of 10%.

As preoperative preparation to prevent intraoperative hemodynamic instability, the alpha-blocker, doxazosin, was started at a dose of 0.5 mg/day, gradually increased to 8 mg/day over a period of about 2 months. Given the 2-stage surgical approach, surgery was first performed for the right adrenal pheochromocytoma. Laparoscopic surgery via a lateral transabdominal approach was performed by placing 4 ports under the right rib arch in the left lateral jackknife position. The mass was easily seen under Gerota fascia and did not invade the surrounding tissues, although a small amount of normal adrenal gland remained at the physiologic attachment site between the liver and the adrenal gland. The tumor was removed along with its capsule, without obvious damage to the capsule. Gross examination of the excised right adrenal gland clearly showed a multinodular lesion measuring 5.0 × 6.0 × 4.5 cm. Histopathologic evaluation of a hematoxylin–eosin stained specimen revealed a pheochromocytoma of the right adrenal gland. In addition, local proliferation of tumor cells was observed, with findings suggestive of venous invasion and partial extracapsular invasion (Fig. 4). Because no thermal damage or other surgical artifacts were identified, these findings were considered to reflect the tumor's biological malignancy rather than surgical manipulation. A retrospective assessment using the GAPP scoring system revealed the following findings: histological pattern, zellballen (0 points); cellularity, moderate (1 point); comedo necrosis, absent (0 points); vascular or capsular invasion, present (1 point); Ki-67 labeling index, 10% (2 points); and catecholamine type, epinephrine (0 points). The total GAPP score was 4 points, corresponding to a moderately differentiated pheochromocytoma.

## Outcome and follow-up

**Figure 5:**
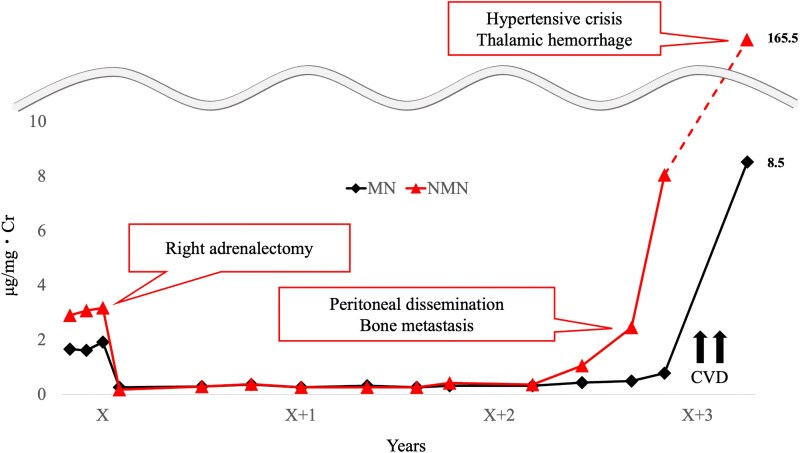
Spot urine levels of metanephrine (MN) and normetanephrine (NMN). Both MN and NMN were elevated at the time of pheochromocytoma diagnosis, but only NMN was elevated at the time of recurrence. MN was also elevated at the time of hypertensive crisis, but it accounted for less than 5% of the total amount of MN and NMN. For reference, the SI conversion factors are as follows: 1 µg MN = 5.07 nmol, and 1 µg NMN = 5.46 nmol. CVD, cisplatin, vincristine, and dacarbazine, Cr, creatinine.

**Figure 6:**
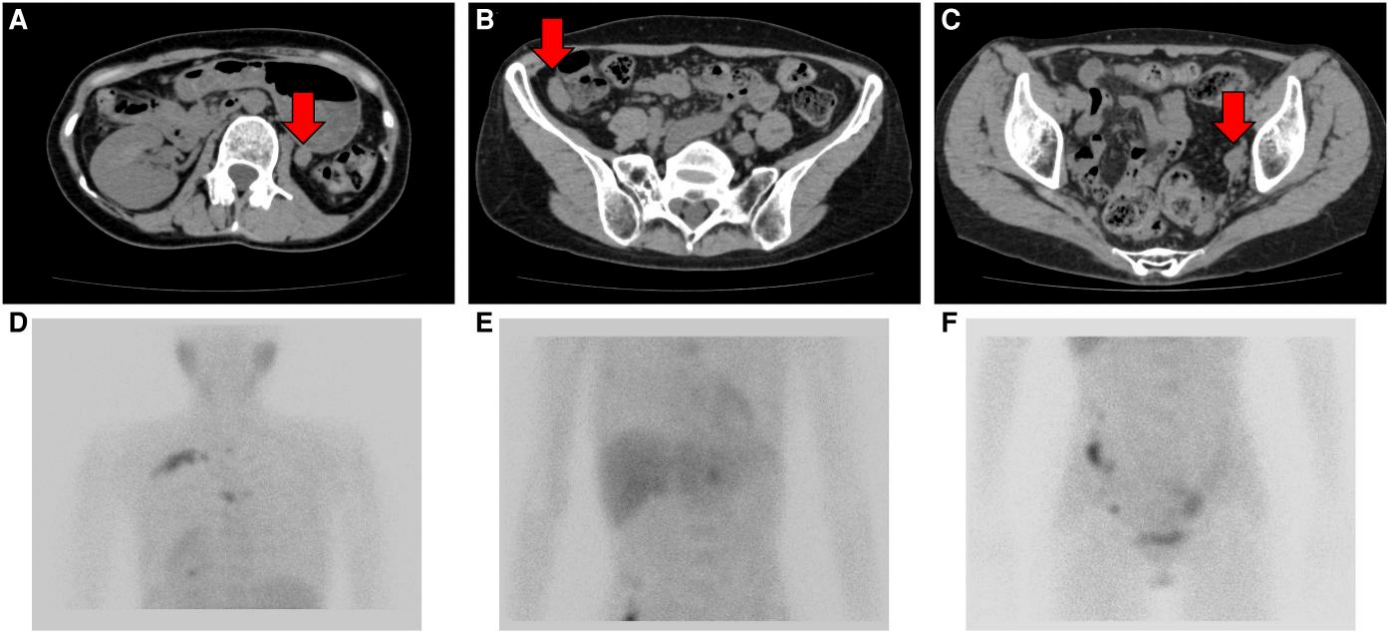
Imaging findings at the time of recurrence. CT showed multiple disseminated nodules in the abdominal cavity (A–C), and ^123^I-meta-iodobenzylguanidine (MIBG) scintigraphy showed increased accumulation in the right ribs and thoracic spine (D) and in the disseminated nodules (E, F).

After resection of the right adrenal pheochromocytoma, a significant decrease in urinary fractionated MN levels was observed, leading us to consider the hormonal activity of the left adrenal tumor to be relatively low; therefore, an active surveillance strategy was adopted to prioritize preservation of adrenal cortical function. One year and 8 months after adrenalectomy, the patient underwent total thyroidectomy and peri-tracheal lymph node dissection for medullary thyroid cancer. Two years and 8 months after adrenalectomy, spot urine MN and NMN showed noradrenergic-type elevations, at 0.49 μg/mgCr and 2.46 μg/mgCr, respectively (Fig. 5), leading to the suspicion of recurrence of pheochromocytoma. CT showed multiple disseminated nodules from the abdomen to the pelvic region, and ^123^I-MIBG scintigraphy showed increased accumulation in the disseminated nodules, left ribs, and thoracic spine at the T2 and T5 levels (Fig. 6). On the other hand, there was no increase in the size of the left adrenal pheochromocytoma. Blood CEA and calcitonin levels were also within the normal range at 1.8 ng/mL (SI: 1.8 μg/L) and <0.50 pg/mL (SI: <0.15 pmol/L), respectively. Therefore, we diagnosed metastasis of adrenal pheochromocytoma. Chemotherapy with a combination of cisplatin, vincristine, and dacarbazine (CVD) or ^131^I-MIBG treatment was proposed, but the patient did not agree, and was instead followed up with continuation of doxazosin (1-4 mg/day). Three years and 1 month after the first surgery, multiple lung, pleural, and liver metastases appeared on CT scan. A 24-hour urine analysis also showed noradrenergic-type elevations of MN and NMN at 1700 μg/day (SI: 8619 nmol/day) and 37 400 μg/day (SI: 204 204 nmol/day), respectively. Although CVD therapy was started, treatment was interrupted at the end of the second course due to the development of disseminated intravascular coagulation. One month later, the patient developed a hypertensive crisis with systolic blood pressure over 200 mmHg, which led to thalamic hemorrhage and death.

## Discussion

Our patient represents a rare case of MEN2A-related pheochromocytoma with metastasis and recurrence. In the condition of hereditary paraganglioma–pheochromocytoma (PPGL), the frequency of metastasis and recurrence is known to vary depending on the causative gene, being as high as 30.7% for succinate dehydrogenase complex subunit B pathogenic variants, and as low as 2.9% for *RET* pathogenic variants observed in MEN2 [5]. A systematic review by Kumar et al [7] also found only 31 cases of metastatic or recurrent MEN2 pheochromocytoma. Hence, the current case is rare from a genetic perspective.

The diagnosis of recurrence in this case was triggered by an increase in urinary NMN levels. The biochemical phenotypes of PPGL are defined as follows: an adrenergic phenotype is defined when plasma free MN levels exceed the upper limit of normal and show an increase accounting for ≥5% of the total increase in plasma free MN and plasma free NMN (or alternatively, based on urinary adrenaline and noradrenaline levels); all other cases are defined as the noradrenergic phenotype [8, 9]. In the present case, this classification was applied using 24-hour urinary MN and NMN measurements as a surrogate, given previous reports demonstrating that relative increases in urinary MN and NMN closely reflect the epinephrine-to-norepinephrine ratio in tumor tissue [10]. According to previous studies, the biochemical phenotypes of pheochromocytoma are considered to reflect the stage of differentiation from neural crest cells to chromaffin cells [11]. The biosynthesis of adrenaline from tyrosine requires tyrosine hydroxylase, dopamine β-hydroxylase, and phenylethanolamine N-methyltransferase. Tumors belonging to the kinase signaling cluster, including those harboring pathogenic variants in *RET*, neurofibromin 1 (*NF1*), transmembrane protein 127, and MYC-associated factor X, generally retain the activity of these enzymes and therefore predominantly exhibit an adrenergic phenotype. Moreover, this biochemical characteristic is often preserved even at the time of metastasis or recurrence [12]. In this context, the observation that a tumor belonging to the kinase signaling cluster demonstrated a noradrenergic phenotype at recurrence is extremely rare. Additionally, bilateral MEN2A-related pheochromocytomas with discordant malignant potential and clinical behavior have not been previously reported. Although it remains unclear whether this phenotypic shift reflects tumor dedifferentiation or indicates the coexistence of additional genetic alterations associated with other molecular clusters, this finding provided an important opportunity to re-evaluate the tumor's biological characteristics in the present case. Further accumulation of detailed case reports and studies is needed to elucidate the biological basis of these tumor phenotypic changes and their clinical implications.

Finally, we discuss the peritoneal dissemination in this case. At the time of the initial tumor excision surgery, histopathological evaluation revealed findings suggestive of capsular invasion, which may have posed a risk of residual microscopic lesions. In addition, intraoperative damage to the tumor capsule can trigger peritoneal dissemination of pheochromocytoma [13], although intraoperative and pathological findings in this case showed no obvious damage to the tumor capsule. Although several reports have described peritoneal dissemination following surgery for pheochromocytoma, there have also been cases—similar to the present case—in which no capsular rupture or intraoperative tumor spillage was documented, yet peritoneal dissemination subsequently occurred [13, 14]. Both the American Endocrine Society guidelines and the Japanese guidelines recommend laparotomy for pheochromocytoma with a large mass (eg, >6 cm) to prevent capsular damage [4, 15]. In this case, the tumor diameter was 4.6 cm on preoperative CT, suggesting that laparoscopic surgery was an acceptable choice. Although the intraoperative and pathological findings in this case did not show evidence of damage to the tumor capsule, the possibility of intraoperative damage to the tumor capsule cannot be completely ruled out, given the subsequent peritoneal dissemination. It has been reported that hand-assisted laparoscopic surgery can be performed for large adrenal masses, although further studies are warranted to confirm this [4, 16].

In summary, we experienced a rare case of metastatic and recurrent MEN2A-related pheochromocytoma. Moreover, the phenotypic shift from an adrenergic to a noradrenergic type was considered a finding that offered an opportunity to re-examine the tumor's biological characteristics in pheochromocytoma. In addition, peritoneal dissemination can occur even in cases without documented capsular rupture, underscoring the importance of gentle tumor handling during surgery.

## Learning points

- The prognosis of MEN2A-related pheochromocytoma is generally good, although some rare cases have an unfortunate turnaround with metastasis and recurrence.
- The biochemical phenotype of pheochromocytoma is considered an important clinical feature reflecting tumor differentiation and the underlying genetic background, and clinicians should pay close attention to this finding in contemporary clinical practice.

## Data Availability

Data sharing is not applicable to this article as no datasets were generated or analyzed during the current study.
